# Beneficial and Sexually Dimorphic Response to Combined HDAC Inhibitor Valproate and AMPK/SIRT1 Pathway Activator Resveratrol in the Treatment of ALS Mice

**DOI:** 10.3390/ijms23031047

**Published:** 2022-01-19

**Authors:** Oluwamolakun Bankole, Ilaria Scambi, Edoardo Parrella, Matilde Muccilli, Roberta Bonafede, Ermanna Turano, Marina Pizzi, Raffaella Mariotti

**Affiliations:** 1 Department of Neurosciences, Biomedicine and Movement Sciences, University of Verona, 37134 Verona, Italy; oluwamolakunoluwatobi.bankole@univr.it (O.B.); ilaria.scambi@univr.it (I.S.); matilde.muccilli@univr.it (M.M.); roberta.bonafede@yahoo.com (R.B.); ermanna.turano@univr.it (E.T.); 2 Department of Molecular and Translational Medicine, Division of Pharmacology, University of Brescia, 25123 Brescia, Italy; e.parrella@unibs.it

**Keywords:** amyotrophic lateral sclerosis, resveratrol, valproate, epigenetics, motor neuron

## Abstract

Amyotrophic lateral sclerosis (ALS) is a fatal adult-onset neurodegenerative disorder. There is no cure and current treatments fail to slow the progression of the disease. Epigenetic modulation in the acetylation state of NF-kB RelA and the histone 3 (H3) protein, involved in the development of neurodegeneration, is a drugable target for the class-I histone deacetylases (HDAC) inhibitors, entinostat or valproate, and the AMP-activated kinase (AMPK)-sirtuin 1 pathway activator, resveratrol. In this study, we demonstrated that the combination of valproate and resveratrol can restore the normal acetylation state of RelA in the SOD1(G93A) murine model of ALS, in order to obtain the neuroprotective form of NF-kB. We also investigated the sexually dimorphic development of the disease, as well as the sex-sensibility to the treatment administered. We showed that the combined drugs, which rescued AMPK activation, RelA and the histone 3 acetylation state, reduced the motor deficit and the disease pathology associated with motor neuron loss and microglial reactivity, Brain-Derived Neurotrophic Factor (BDNF) and B-cell lymphoma-extra large (Bcl-xL) level decline. Specifically, vehicle-administered males showed earlier onset and slower progression of the disease when compared to females. The treatment, administered at 50 days of life, postponed the time of onset in the male by 22 days, but not in a significant way in females. Nevertheless, in females, the drugs significantly reduced symptom severity of the later phase of the disease and prolonged the mice’s survival. Only minor beneficial effects were produced in the latter stage in males. Overall, this study shows a beneficial and sexually dimorphic response to valproate and resveratrol treatment in ALS mice.

## 1. Introduction

Amyotrophic lateral sclerosis (ALS), also known as Lou Gehrig’s disease, is a fatal progressive neurodegenerative disease that affects nerve cells, responsible for controlling voluntary muscle movement in the brain (upper motor neurons) and spinal cord (lower motor neurons) leading to weakness, loss of motor function, muscle atrophy and eventually death due to respiratory failure [1]. ALS is the most common form of motor neuron disease [2], with about 90% of all ALS cases being sporadic, while the other 10% are familial, and about 15–20% of all familial cases are caused by a mutation in the superoxide dismutase-1 glycine 93 to alanine gene (SOD1 G93A) [3]. Furthermore, studies have reported genetic and clinical differences in the onset and progression of the disease in the male and female ALS mice model, as well as in clinical patients [4]. SOD1(G93A) female mice with B6SJL background generally experience a delay in disease onset and an increase in survival time compared with their male counterparts [5]. In terms of genetic differences, SOD1(G93A) female mice often show a delay in mitochondrial dysfunction due to decreased mitochondria oxidative phosphorylation associated with protein oxidative damage, compared to their male SOD1(G93A) mice. This delayed mitochondrial dysfunction has been associated with higher estrogen levels in females [6]. In clinical patients, it is generally reported that men are at higher risk of developing ALS with a male-to-female ratio of 1:2 [7,8]. Moreover, the ALS symptomatology is different in men and women and depends on which neurological region or motor level is affected: there is a predominance of earlier limb onset in males and later bulbar onset in females [9]. However, survival is similar in the two sexes. Many factors have been implicated in the degeneration of motor neurons (MN), such as glutamate excitotoxicity, mitochondrial dysfunction, inflammatory response and oxidative stress [10]. More recently, transcriptional dysfunction, a mechanism which involves aberrations of the molecular machinery that regulates gene expression, has been implicated in MN degeneration [11]. The level at which these aberrations in gene expression occur is known as epigenetic marks. Among these marks, acetylation of histone, the protein that makes up the chromatin component, has been characterized as an important mechanism involved in increased DNA transcriptional activity [12]. Defect in this histone homeostasis (acetylation and deacetylation) has been implicated in several neurodegenerative diseases, including ALS [11,13,14]. The status of histone homeostasis strongly depends on the activity of a family of enzymes known as histone deacetylases (HDACs), which catalyze histone deacetylation in the chromatin structure and have been implicated in several cellular processes, such as cell death and stress response [15]. HDACs also play an important role in regulating the acetylation of non-histones proteins, such as the nuclear factor kappa-light-chain enhancer of activated B cells (NF-κB). NF-κB is a transcription factor involved in a variety of physiological processes in the nervous system [16], but also in pathological mechanisms associated with neurodegeneration [17,18]. Five subunits compose the NF-κB family of proteins: RelA (p65), RelB, c-Rel, p50 and p52. NF-κB neuroprotective or neurotoxic roles depends on the subunits forming the transcriptional active dimmer [19,20]. The acetylation state of the RelA subunit can promote both neuroprotective and neurotoxic actions [21,22]. In particular, we previously showed that a specific aberrant acetylation pattern of RelA, characterized by a reduced level of total acetylation, but site-specific acetylation at the lysine 310, RelA Ac(K310), triggers the expression of pro-apoptotic genes in brain ischemia [21].

Several studies suggested a role of RelA in ALS pathogenesis [23]. RelA subunit has been shown to be upregulated in cellular [24,25,26] and animal models of ALS [27,28]. Of note, RelA levels were elevated also in the spinal cord of ALS patients [28,29,30]. Importantly, we recently demonstrated that the pro-apoptotic acetylation state of RelA, consisting of global lysine deacetylation and an increased lys 310 acetylation, was evident in the lumbar spinal cord of SOD1(G93A) mice [31].

A body of evidence has indicated that pharmacological inhibition of HDACs with drugs such as butyrate and vorinostat helps to improve cell survival by promoting acetylation of histone, gene transcription and protein synthesis in murine models of ischemic stroke and a murine model of ALS [32]. Combined drug treatment with the class-I HDAC inhibitor valproate (VPA) [33,34] and lithium has been shown to improve motor function, delay disease onset and prolong the life span of SOD1(G93A) mice [35]. Furthermore, pharmacological stimulation of the class-III HDAC sirtuin 1 (SIRT1), and the AMP-activated kinase (AMPK) by resveratrol (RESV) has been shown to protect against motor neuron death and improve survival in the SOD1(G93A) ALS mice model [36].

We previously reported that the combined administration of low doses of the class-I HDAC inhibitor entinostat (MS-275) and RESV promote the normalization of the RelA acetylation state and revert the pathological histone H3 deacetylation in brain ischemia as well as ALS animal models [31,37]. The combination MS-275 and RESV corrected the pathological acetylation state of RelA by, respectively, enhancing the RelA general acetylation and reducing the acetylation at the lys 310 via SIRT1 activation [31,37]. Moreover, RESV activated the AMPK pathway, leading to an increase of acetyl-CoA and the subsequent activation of histone acetyl-transferases (HATs), the enzymes responsible for the acetylation of both RelA and H3 histones. By the generation of NAD+, the fundamental cofactor for class III HDACs [38], AMPK could also sustain SIRT1 activation. In SOD1(G93A) mice, the correction of RelA acetylation by MS-275 and RESV protected MN from neurodegeneration, activated the anti-apoptotic Bcl-xL (B-cell lymphoma-extra large) and neurotrophic BDNF (Brain-Derived Neurotrophic Factor), delayed symptoms onset and extended lifespan [31].

MS-275 is currently in clinical trials for the treatment of different types of cancer but has not yet been approved for clinical use [39]. For translational purposes, the present study investigated the efficacy of MS-275 replacement with valproate (VPA), an antiepileptic drug also reported to be a class I HDAC blocker. As a class I HDACs inhibitor, VPA could represent an alternative to MS-275, with the advantage of being suitable for translational application in clinical trials for ALS, as it is already being routinely used to treat epileptic and bipolar patients [33]. Furthermore, VPA has been reported to be neuroprotective in both animal and cellular models of brain ischemia [40,41]. Resveratrol (trans-3,4′,5-trihydroxystilbene) is a natural polyphenol endowed with multiple beneficial properties, including neuroprotective effects [42]. Of note, we recently showed that VPA and RESV can synergize in promoting neuroprotection in cellular and animal models of brain ischemia by restoring the RelA physiological acetylation state and reverting the histone H3 hypoacetylation [43].

Based on these previous findings, we investigated, for the first time, the effect of the combined treatment of VPA with RESV in ALS SOD1(G93A) male and female mice. The combined administration of RESV and VPA postponed the time of onset in males, while in the female counterpart it reduced symptom severity, especially in the late phase of the disease, and prolonged the survival. The combined drugs were able to restore the correct RelA and histone 3 acetylation state in the lumbar spinal cord of mice in both sexes. This effect was associated with an overall neuroprotective effect on motor neurons and a reduced microglial immunoreactivity in the lumbar spinal cord.

## 2. Results

### 2.1. Untreated SOD1(G93A) Female Mice Show Delayed Decline in Motor Performance Compared to Male Counterparts

**Figure 1:**
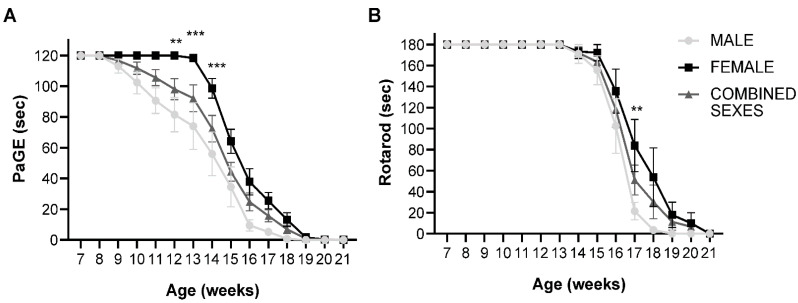
PaGE and rotarod test of untreated male, female and combined sexes: (**A**) PaGE test showing grip endurance of untreated SOD1(G93A) animals. There was a significant difference between male and female untreated groups at week 12 (*p* = 0.0038), week 13 (*p* = 0.0003) and week 14 (*p* = 0.0006), where the female animals displayed significantly better performance compared to the male mice (n = 10 male, 10 female); (**B**) Rotarod test showing motor coordination of untreated SOD1(G93A) animals. A significant difference between male and female mice was observed, with the female untreated mice showing significantly better performance at week 17 (*p* = 0.0025) compared to male animals. Results were analyzed by two-way ANOVA followed by the Bonferroni multiple comparisons test. ** *p* < 0.005; *** *p* < 0.0005. Data in all graphs are expressed as mean ± SEM.

To determine the difference in the clinical onset and rate of disease progression in both male and female SOD1(G93A) mice, the animals were divided into two groups based on their sex. Both male and female animals were subjected to two motor tests. Paw grip endurance test (PaGE), which was used to examine the grip strength of the animals and to establish the onset of the disease, and the rotarod test to examine motor coordination in the animals. In the PaGE test, the female animals exhibited a delay in the decline of motor performance compared to male mice. Female animals began to show a decrease in motor performance at week 14 compared to male mice that experienced a decline in motor performance as early as week 9. A significant difference between the two sexes was observed at week 12 (*p* = 0.0038), week 13 (*p* = 0.0003) and week 14 (*p* = 0.0006), with female animals showing better performance as opposed to male mice (Figure 1A). Similar results were obtained in the rotarod test, where the female animals performed significantly better than their male counterparts at week 17 (*p* = 0.0025), indicating that female SOD1(G93A) mice with B6SJL background generally show a delay in clinical onset compared to male mice (Figure 1B).

### 2.2. VPA and RESV Improve Motor Performance in SOD1(G93A) Mice

**Figure 2:**
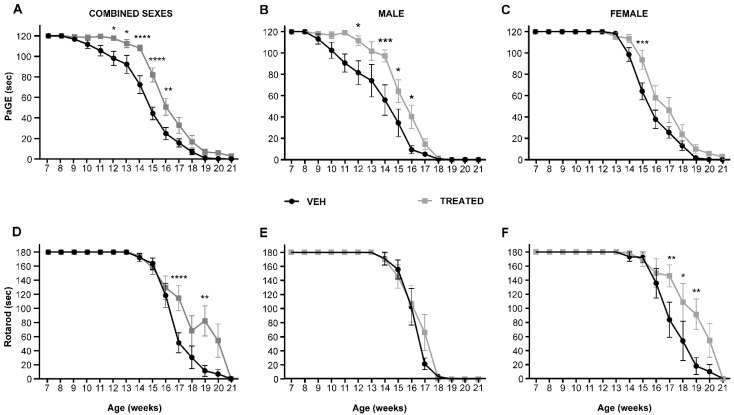
PaGE and rotarod tests in combined sexes, male and female SOD1(G93A) mice treated with pharmacological combinations of VPA and RESV (TREATED), or untreated (VEH): (**A**) PaGE test for combined sex animals showed that in the TREATED animals, a significant improvement in motor performance was observed at week 12 (*p* = 0.0382), week 13 (*p* = 0.0272), week 14 (*p* < 0.0001), week 15 (*p* < 0.0001) and week 16 (*p* = 0.010) compared to the VEH (n = 20 VEH, 24 TREATED); (**B**) Male SOD1(G93A) mice showed a significant improvement in the TREATED group compared with VEH at week 12 (*p* = 0.0430), week 14 (*p* = 0.0007), week 15 (*p* = 0.0480) and week 16 (*p* = 0.0327; n = 10 VEH, 12 TREATED); (**C**) The female mice displayed a significant improvement at week 15 in the TREATED versus the VEH group (*p* = 0.0009; n = 10 VEH, 12 TREATED); (**D**) The motor coordination test of the combined sexes showed a significant improvement in motor performance of the TREATED mice compared with the VEH animals at week 17 (*p* < 0.0001) and week 19 (*p* = 0.0021; n = 20 VEH, 24 TREATED); (**E**) Rotarod test in the SOD1(G93A) male mice showed no statistical significance in the TREATED versus the VEH animals (n = 10 VEH, 12 TREATED); (**F**) A significant improvement was observed in the TREATED female SOD1(G93A) mice at week 17 (*p* = 0.0040), week 18 (*p* = 0.0262) and week 19 (*p* = 0.0077) with respect to the VEH group, (n = 10 VEH, 12 TREATED). Results were analyzed by two-way ANOVA followed by the Bonferroni multiple comparisons test. * *p* < 0.05; ** *p* < 0.005; *** *p* < 0.0005; and **** *p* < 0.0001. Data in all graphs are expressed as mean ± SEM.

To evaluate the effect of the combined drugs (RESV and VPA) on the motor performance of the SOD1(G93A) mice in combined sexes—male and female mice—the animals were divided into the treated group (TREATED) for the administration of a combined treatment of RESV (136 μg/kg/day) and VPA (40 μg/kg/day), and the vehicle group (VEH). Both groups of animals began treatment at post-natal week 7, close to the onset stage, as detected through the PaGE test, and continued the daily treatment until they were sacrificed at the end stage of the disease. The dose of the drug to be administered was regulated every week based on the body weight of the animals. The body weight and motor tests PaGE and rotarod were assessed every week. Regarding the grip persistence of the animals, results revealed an overall improvement in motor performance of animals in the TREATED group compared to the VEH group. Specifically, in the combined sexes, the drug treatment significantly delayed the loss of motor function at week 12 (*p* = 0.0382), week 13 (*p* = 0.0272), week 14 (*p* < 0.0001), week 15 (*p* < 0.0001) and week 16 (*p* = 0.010) (Figure 2A). Interestingly, when divided into different sexes, the male mice treated with the drugs showed a higher significant improvement in motor performance at week 12 (p= 0.0430), week 14 (*p* = 0.0007), week 15 (*p* = 0.0480) and week 16 (*p* = 0.0327) compared to the VEH group (Figure 2B), while the female animals only showed a significant improvement at week 15 (*p* = 0.0009) compared to VEH group (Figure 2C). The rotarod test was also performed to examine motor coordination in the TREATED and VEH animals. In the combined sexes, it was observed that animals in the TREATED group showed a significant improvement in motor coordination at week 17 (*p* < 0.0001) and week 19 (*p* = 0.0021) compared to the VEH group (Figure 2D). Surprisingly, the male animals only showed a trend in the improvement of motor function after drug treatment without any statistical significance (Figure 2E). The female animals in the TREATED group showed better performance in the rotarod test with statistical significance at week 17 (*p* = 0.0040), week 18 (*p* = 0.0262) and week 19 (*p* = 0.0077) (Figure 2F). An overall significant improvement in motor performance was recorded in the TREATED groups compared to the control.

### 2.3. Combined Administration of VPA and RESV Showed a Postponement of Disease Onset and Survival in Sex-Depending Manner in SOD1(G93A) Mice

**Figure 3:**
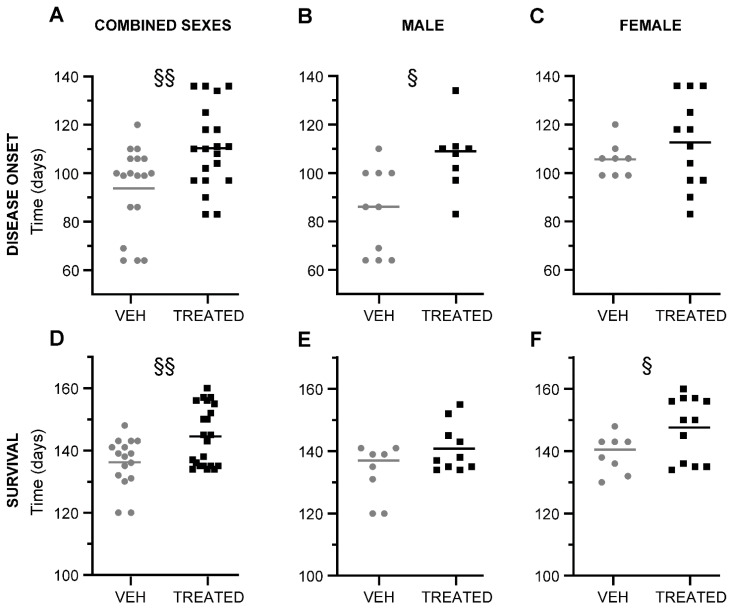
Disease onset and survival of combined sexes, male and female SOD1(G93A) mice in TREATED and VEH group: (**A**) The graph shows the disease onset of combined sex mice in the TREATED and VEH group. A significant delay in disease onset was observed in the TREATED animals compared to the VEH mice (*p* = 0.0055; n = 18 VEH, 20 TREATED); (**B**) The male mice in the TREATED group showed a statistically significant delay of disease onset compared with the VEH, (*p* = 0.0107; n = 10 VEH, 8 TREATED); (**C**) In the female animals, a trend was observed in the delay of disease onset of animals treated with the drugs, but was without statistical significance (*p* = 0.3294; n = 8 VEH, 12 TREATED); (**D**) The graph shows the rate of survival of combined sexes in the TREATED group compared to the VEH. The treated mice showed a significant improvement in survival rate compared to the VEH (*p* =0.0074; n = 16 VEH, 22 TREATED); (**E**) Male animals in the TREATED group displayed no significance in survival rate (*p* = 0.0702; n = 8 VEH, 10 TREATED); (**F**) In the female mice, an improvement in the lifespan of treated animals was observed with respect to the VEH mice (*p* = 0.0497; n = 8 VEH, 12 TREATED). Statistical analysis was performed with Log-rank (Mantel–Cox) test. Data in all graphs are expressed as mean ± SEM. § *p* < 0.05; §§ *p* < 0.005.

To examine the effect of the combined drug treatment in the ALS murine model SOD1(G93A), the clinical onset of the disease, as well as the life span were monitored in both the TREATED and VEH groups of the combined sexes, male and female mice. We first evaluated the disease onset in the combined sexes. Here, we observed that there was a significant delay in the disease onset of TREATED animals (*p* = 0.0055), with respect to the VEH mice. The TREATED mice experienced disease onset at approximately 110 days after birth, while animals in the VEH group displayed earlier disease onset at 94 days. The mean difference between the two groups was recorded as 16 ± 5.5 days (Figure 3A). Surprisingly, the further sex-related analysis revealed that the male animals treated with the drugs exhibited a significant delay in the onset of the disease (107 days) compared to the VEH animals (84 days). A delay of 22 ± 7.8 days was observed between the two groups (*p* = 0.0107) (Figure 3B). The female animals only showed 7 days delay in clinical onset when treated with the drugs, without any statistical significance (*p* = 0.3294) (Figure 3C). The survival rate of the animals was also taken into consideration. In the combined sexes (Figure 3D), a significant increase in survival time was observed in the TREATED group (145 days) with respect to the VEH mice (136 days). The mean difference between the two groups was 8.3 ± 2.9 days (*p* = 0.0074). Specifically, the male animals in the TREATED group did not show any significant improvement in the rate of survival compared to the VEH group, although a trend was observed (*p* = 0.0702) (Figure 3E). Interestingly, treated females showed a statistically significant improvement in survival rate (148 days) (*p* = 0.0497), as opposed to the VEH female mice (139 days). A mean difference of 8.5 ± 4 days in the survival rate was observed (Figure 3F).

### 2.4. Drug Treatment Protects Lumbar Spinal Cord from Neurodegeneration

**Figure 4:**
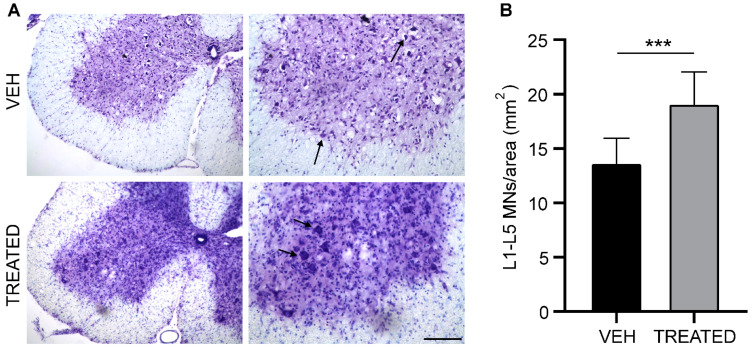
The MN count in the lumbar tract of the spinal cord (L1- L5): (**A**) Nissl staining on the L3 lumbar segment of the spinal cord at the end stage of the disease in VEH and TREATED groups. The arrow shows stained MN; (**B**) The graph shows the MN number/area in the L1-L5 tract of both the TREATED and VEH groups. In the TREATED group, there was a significant increase in MN survival compared to the VEH group (*p* = 0.0002; n = 10 VEH, 12 TREATED). Images on the left panel have a magnification of 10×, which images on the right panel have a magnification of 20× (scale bar = 100 μm). Data were analyzed by the Student *t*-test, expressed as mean ± SEM. *** *p* < 0.0005.

To evaluate the neuroprotective effect of RESV and VPA in SOD1(G93A) mice, Nissl staining and the stereological count of motoneurons (MN) in the lumbar spinal cord were performed at the end stage of the disease. The MN population per area in the ventral horn of the lumbar tract (L1–L5) was analyzed in both the TREATED and VEH groups. In the combined sexes, a 40% increase in MN numbers was observed in TREATED mice compared to animals in the VEH group (*p* = 0.0002) (Figure 4B). Furthermore, we examined the MN population in both male and female SOD1(G93A) mice and obtained similar results. Both male and female mice in the TREATED group showed a significant increase in the MN population compared to their VEH counterparts (57% and 60% respectively, see Supplementary Materials, Supplementary Data1, Figure S1). The increase in the MN population was also associated with a significant decrease in microglia activation in the TREATED group of male, female and combined sexes (33%, 38% and 31%, respectively) (see Supplementary Materials, Supplementary Data1, Figure S2). These results revealed a protective effect of the combined drug treatment on lumbar MN (Figure 4).

### 2.5. VPA and RESV Restore Physiological RelA Acetylation State in Lumbar Spinal Cord of SOD1(G93A) Mice

**Figure 5:**
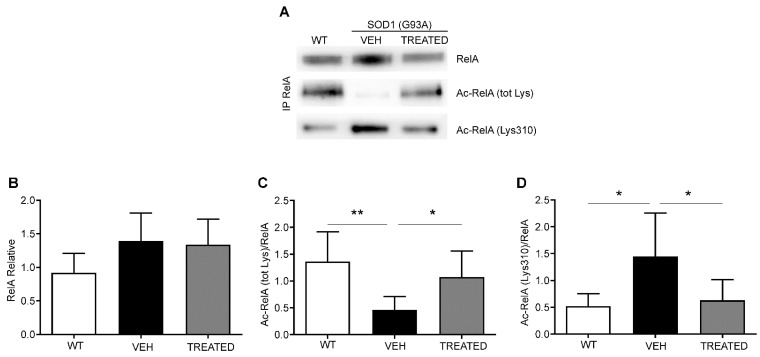
Immunoprecipitation and densitometric analysis of RelA acetylation in combined sex mice: (**A**) Representative image of immunoprecipitation and Western blot analysis of nuclear RelA (65 kDa), total lysine acetylation of RelA (Ac-RelA (tot lys)) and acetylation of lysine 310 (Ac-RelA (lys310)); (**B**) Densitometric analysis shows a trend in the increase of RelA in the VEH and TREATED groups compared to the WT; (**C**) Ac-RelA (tot lys) acetylation was statistically reduced in the VEH group compared to the WT (*p* = 0.0035) and TREATED group (*p* = 0.0461); (**D**) Ac-RelA (Lys 310) was significantly increased in the VEH group compared to the WT (*p* = 0.0216), and to the TREATED group (*p* = 0.0439). (n = 6 WT, 10 VEH, 12 TREATED) Results were analyzed by one-way ANOVA, followed by Tukey’s multiple comparisons test. Graphs are shown as mean ± SEM. * *p* < 0.05; ** *p* < 0.005. Full length blots are shown in Supplementary Materials in Supplementary Data 2 Figure S7.

Immunoprecipitation and Western blot analyses were performed to assess the acetylation state of the RelA protein from the nuclear fraction of the lumbar spinal cord of WT, VEH and TREATED SOD1(G93A) male and female mice at end stages of the disease (Figure 5A). A trend in the increase of RelA nuclear level appeared in SOD1(G93A), VEH and TREATED groups, when compared to WT mice, though it did not reach a statistical significance (Figure 5B). Mice in the VEH group showed a significant reduction in the total acetylation of immunoprecipitated RelA compared to the WT (*p* = 0.0035). The treatment led to a significant increase in the total acetylation state of RelA (*p* = 0.0461) (Figure 5C). Immunoprecipitation was also performed to examine the site-specific acetylation of RelA at lysine 310. Results obtained showed a significant increase in the Lys 310 acetylation in the VEH group compared to WT (*p* = 0.0216), followed by a significant restoration of the acetylation state in animals that received the treatment (*p* = 0.0439) (Figure 5D). The animals were also specifically divided into male and female groups and the RelA acetylation was examined. Similar results were obtained, and no difference was observed between the acetylation state of male and female animals (see Supplementary Materials, Supplementary Data1, Figure S3). As reported in previous studies [31], we demonstrated a general alteration of the acetylation state of RelA in SOD1(G93A) mice, and the combined drug treatment resulted in a restoration of the physiological RelA acetylation state evident in WT animals.

### 2.6. RESV Increases Phosphorylation of AMPK in the Lumbar Spinal Cord of SOD1(G93A) Mice

**Figure 6:**
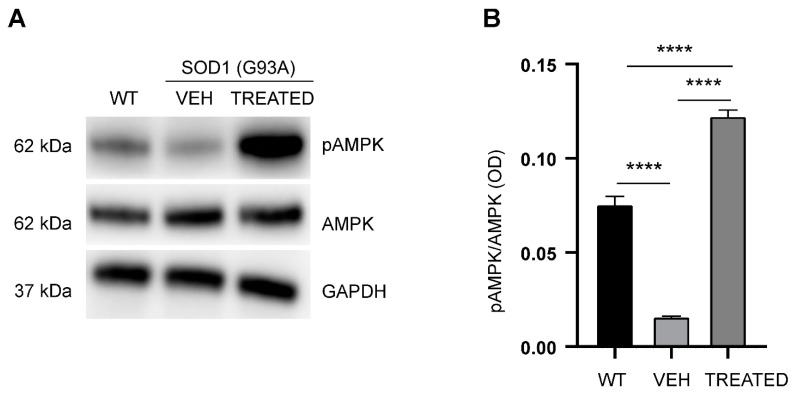
Analysis of phosphorylation state of AMPK in the experimental groups: (**A**) Representative Western blot showing the phosphorylation of AMPK; (**B**) Phosphorylation state of AMPK was significantly reduced in the VEH group compared with the WT (*p* < 0.0001). In the TREATED group, the phosphorylation state of AMPK was significantly increased versus the VEH (*p* < 0.0001) and the WT (*p* < 0.0001) groups (n = 6 WT, 10 VEH, 12 TREATED). Results were analyzed by one-way ANOVA, followed by Tukey’s multiple comparisons test. Data are shown as mean ± SEM. **** *p* < 0.0001. Full length blots are shown in Supplementary Materials in Supplementary Data 2 Figure S8.

To further examine the effect of RESV on the protein target AMPK, the phosphorylated state of AMPK (pAMPK) was analyzed using the cytoplasmic protein fraction of the lumbar spinal cord of WT, VEH and TREATED mice, at the end stage of the disease (Figure 6A). A significant decrease in the phosphorylation state of Tr172 of pAMPK was observed in VEH animals compared to WT (*p* < 0.0001) (Figure 6B). However, the drug treatment resulted in a subsequent significant increase in phosphorylation of AMPK compared with the VEH (*p* < 0.0001), and even above the WT (*p* < 0.0001). Regarding the sex-specific study, similar results were obtained in both male and female SOD1(G93A) mice, indicating no sex-specific differences in AMPK phosphorylation at the end stage of the disease (see Supplementary Materials, Supplementary Data1, Figure S4).

### 2.7. Increased Neurotrophic Factor BDNF and Anti-Apoptotic Bcl-xL Protein Levels in the Lumbar Spinal Cord of SOD1(G93A) Mice

**Figure 7:**
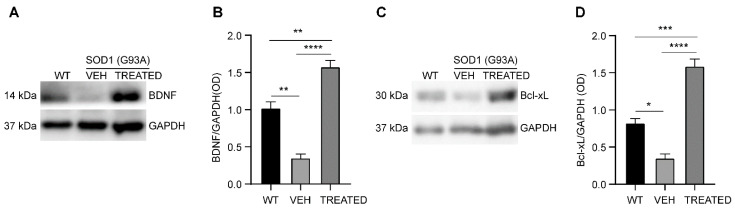
Densitometric analysis of the expression of neurotrophic factor, BDNF and anti-apoptotic factor Bcl-xL normalized to GAPDH: (**A**) Representative image of Western blot assay showing the expression of BDNF (14 kDa) and GAPDH (37 kDa); (**B**) BDNF level was reduced in the VEH animals compared to the WT, with a significance of *p* = 0.0019. The treatment significantly increased the expression of BDNF compared to the VEH (*p* < 0.0001) and the WT groups (*p* = 0.0049); (**C**) A representative image of Bcl-xL (30 kDa) and GAPDH (37 kDa) Western blot; (**D**) The anti-apoptotic factor Bcl-xL was reduced in the VEH group compared to the WT, with a significance of *p* = 0.0176. Pharmacological treatment significantly increased Bcl-xL protein level when compared to either the VEH (*p* < 0.0001) or the WT (*p* = 0.0001) groups. The same number of animals (n = 6 WT, 10 VEH, 12 TREATED) used for AMPK analysis was also used for the BDNF and Bc-xL studies. Results were analyzed by one-way ANOVA, followed by Tukey’s multiple comparisons test. Data are shown as mean ± SEM. * *p* < 0.05; ** *p* < 0.005; *** *p* < 0.0005; and **** *p* < 0.0001. Full length blots are shown in Supplementary Materials in Supplementary Data 2 Figure S9.

The level of brain-derived neurotrophic factors (BDNF) and Bcl-xL protein was examined in the cytoplasmic protein fraction of the lumbar spinal cord of WT, VEH and TREATED SOD1(G93A) mice at the end stage of the disease (Figure 7A). It was observed that the neurotrophic factor BDNF was significantly reduced in the VEH group compared to the WT (*p* = 0.0019). The drug treatment resulted in a significant increase in the BDNF levels in the TREATED group with respect to the VEH (*p* < 0.0001) and the WT (*p* = 0.0049), (Figure 7B). Moreover, a reduction in the anti-apoptotic factor Bcl-xL protein, was observed in the VEH animals compared to the WT (*p* = 0.0176), while the combined drug treatment significantly improved the expression of Bcl-xL protein with respect to the VEH (*p* < 0.0001), and WT as well (*p* = 0.0001) (Figure 7C, D). Similar results were obtained both in male and female animals (see Supplementary Materials, Supplementary Data1, Figure S5).

### 2.8. Similar Restoration of H3 Acetylation State in the Lumbar Spinal Cord of SOD1(G93A) Male and Female Mice

**Figure 8:**
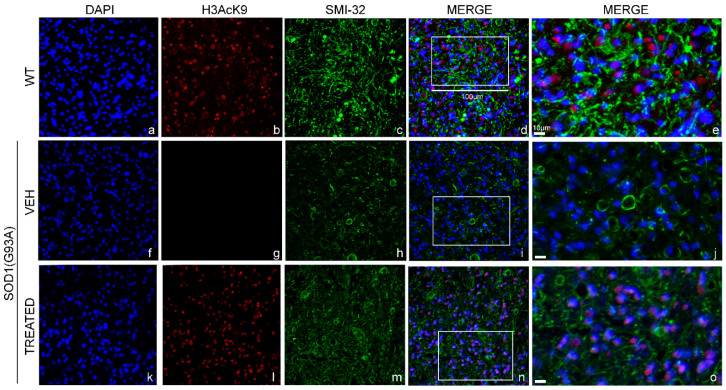
Histone 3 acetylation in the lumbar spinal cord of male WT and SOD1(G93A) mice: The figure panel shows the different acetylation states of lysine 9 of histone 3 in the lumbar spinal cord of wild type (WT) mice, VEH and TREATED SOD1(G93A) groups. The nuclei were stained in blue with DAPI (**a**,**f**,**k**). The acetylation state of histone 3 was identified by the H3AcK9 antibody in red (**b**,**g**,**l**), while the motor neurons (MN) was detected by identifying the antibody neurofilament H with the SMI-32 antibody in green (**c**,**h**,**m**). The acetylation state of histone 3 was drastically reduced in the VEH group (**g**) compared to WT animals (**b**). The treatment with RESV and VPA led to a restoration of the acetylation of histone 3 in the TREATED group (**l**), (n = 3 WT, 5 VEH, 6 TREATED). Figure (**d**,**i**,**n**) shows the superimposed images of DAPI, SMI-32 and H3AcK9 in the WT, VEH and TREATED groups, respectively. Magnification of 20×, scale bar = 100 μm (**a**–**d**,**f**–**i**,**k**–**n**). The figure panel (**e**,**j**,**o**) shows the highlighted area in (**d**,**i**,**n**) at a higher magnification of 40×, scale bar = 10 μm.

Immunofluorescence staining was performed to examine the effect of epigenetic drugs on the acetylation state of Lysine 9 of histone 3 (H3AcK9) (Figure 8b,g,l), co-localized with the nuclei (DAPI) (Figure 8a,f,k) in the lumbar MN (SMI-32) (Figure 8c,h,m) of WT, VEH and TREATED SOD1(G93A) male mice. Qualitative analysis of immunofluorescence images revealed a decrease in the acetylation of H3 in the VEH group (Figure 8g) compared to matched WT (Figure 8b). Furthermore, we observed that the treatment led to a restoration of H3 acetylation (Figure 8l), compared to animals that did not receive any treatment (VEH) (Figure 8g). The level of H3 restoration was similar to what was observed in WT animals (Figure 8b). Noteworthy, similar results were observed also in the female SOD1(G93A) mice, proving that VPA was able to regulate the activity of HDACs class-I and restore the H3 acetylation state in both sexes (see Supplementary Materials, Supplementary Data1, Figure S6).

## 3. Discussion

This study shows that treatment of SOD1(G93A) mice with the combination of VPA and RESV promoted a significant improvement in motor performances, the delay of disease onset, and longer survival.

We observed a sexually dimorphic behavior of the ALS mouse model in response to the treatment. In males, the combination VPA and RESV exerted a beneficial action in the early phase of the disease, resulting in an onset delay, but not in the later phase. Conversely, in females, the drug’s effect appeared more evident in the later phase of the disease, when we detected a reduction of motor deficits and a significant extension of the survival.

The beneficial effect of the treatment on motor performances was coupled to a protective effect on MN and a reduced microglial immunoreactivity in the lumbar spinal cord. Moreover, in accordance with our previous study employing the combination MS-275 and RESV in SOD1(G93A) mice, we observed an increased expression of NF-κB target genes, the anti-apoptotic protein Bcl-xL and the neurotrophic factor BDNF in the lumbar spinal cord of TREATED animals [31]. Previous studies reported that the enhancement of Bcl-xL levels protected MN, quenched microglial activation, delayed disease onset, extended survival, and ameliorated motor deficits in SOD1(G93A) mice [44]. Interestingly, we previously showed that MS-275 and RESV drive RelA recruitment and H3 acetylation to the Bcl-xL promoter, allowing the expression of the anti-apoptotic factor [37].

Similarly, BDNF promoted MN differentiation and survival [45]. A protective role of BDNF has been observed in a cellular model of ALS [46]. Furthermore, the use of recombinant human BDNF has been shown to improve motor functions in the Wobbler mouse model of ALS [47]. Of note, BDNF was able to promote the anti-apoptotic effect by upregulating Bcl-xL expression in vivo [48], indicating crosstalk between the two pathways.

In accordance with our initial hypothesis, the combination of VPA and RESV reverted the mismatch of the RelA acetylation state in the spinal cord of SOD1(G93A) mice by improving the RelA general acetylation and reducing the acetylation at Lys 310. The drug combination also dramatically enhanced the activation of AMPK, therefore supporting the HATs activity necessary to restore the proper RelA acetylation [37]. Moreover, the treatment reverted the histone H3 deacetylation observed in the lumbar spinal cord of SOD1(G93A) mice. Taken together, these results indicate that in the SOD1(G93A) mouse model, VPA synergizes with RESV in a similar fashion and with a similar mechanism as was seen with the combination MS-275 and RESV [31].

A differential effect of the treatment between male and female SOD1(G93A) mice was observed in terms of disease onset and survival, but not at a molecular level. It is important to notice that the pathology analysis was performed on tissue samples collected from animals at the disease endpoint. It is plausible that possible differences among sexes, occurring earlier in the disease progression and that we missed, could have contributed to the observed dimorphic response to the drugs. Alternatively, the dimorphic response to the drugs could rely on mechanisms alternative to those investigated in this study, which remain to be clarified. A possible condition leading to the dimorphic response to the treatment may be the different progression of the disease observed in males and females. This study confirmed a sexual dimorphism in the SOD1(G93A) mouse model of ALS [4]. In accordance with previous reports, SOD1(G93A) male mice showed earlier motor deficit symptoms and disease onset, as well as slower disease progression than females [5,49,50,51,52]. Hypothesizing a beneficial effect of the treatment in mild and moderate stages of the pathology, it is conceivable that the drug’s efficacy was more evident in males only in the first phase of the disease, when symptoms were milder and progression slow.

Another intriguing hypothesis is that the drugs, besides the investigated effect on NF-κB/RelA and histone acetylation, could specifically act on sex-related molecular targets. The molecular bases of sex-dimorphism in ALS are not yet fully understood [4]. Several factors have been suggested to be involved in ALS sex differences, including *C9ORF72* genetic factor [53], mitochondrial function [6] and sex hormones. In particular, female sex steroids have been proposed to play an important beneficial role in ALS, exerting both neuroprotective and anti-inflammatory actions [4]. Interestingly, estrogen receptors (ERs) and aromatase, the enzyme responsible for estrogen synthesis, are expressed in the lumbar spinal cord of adult mice, suggesting that circulating and locally synthesized estrogens could mediate neuroprotection in spinal cord MN. Estrogen efficiently improved locomotor function when administered in a mouse model of spinal cord injury or ALS [54]. We speculate that VPA and RESV could potentiate female sex steroids in the SOD1(G93A) mouse model, resulting in a sustained function of degenerating MN. RESV is structurally similar to natural and synthetic estrogens and is endowed with estrogenic activity [55]. This occurs via multiple mechanisms, including a direct action on ERs, modulation of steroidogenesis and inhibition of estrogens metabolism that potentially strengthen estrogen action [55]. Moreover, studies on different experimental models indicated that SIRT1 and AMPK, two proteins activated by RESV, are necessary for modulating ER-signaling pathways [56,57,58]. Likewise, VPA has been shown to upregulate ERs in cancer and heart tissues [59,60,61].

Both VPA and RESV have been individually tested against ALS, with mixed results [23]. Oral treatment of SOD1(G93A) male mice with VPA at the antiepileptic dose of 500 mg/kg/day increased lifespan without postponing disease onset [62]. In another study, VPA orally administered at antiepileptic dose slowed down MN death but failed in improving lifespan in SOD1(G93A) mice of both sexes (analyzed together) [63]. Intraperitoneal administration of VPA twice a day at the dose of 300 mg/kg delayed disease onset, ameliorated motor deficits and prolonged lifespan in SOD1(G93A) male and female mice [35]. VPA injected to SOD1(G86R) male mice (250 mg/kg/day, i.p.) postponed disease onset without increasing lifespan [64]. VPA has also been tested in a phase II clinical trial with a dose used in epilepsy (1500 mg daily), but it did not provide any beneficial effect on survival or disease progression in ALS patients [65].

Oral administration of RESV (160 mg/kg/day) to SOD1(G93A) male and female mice delayed disease onset, extended life span and reduced MN degeneration [36]. A lower dose of RESV (25 mg/kg/day) administered with the diet did not promote any beneficial effect in SOD1(G93A) female mice [66]. On the other hand, intraperitoneal injections of RESV twice a week at the dose of 20 mg/kg delayed disease onset, extended survival and preserved MN survival in SOD1(G93A) male mice [67].

Although some of the studies listed above provided beneficial results sometimes comparable to the ones reported in this research, the doses of VPA and RESV administrated individually were several folds higher than the dosage of the molecules in combination. Therefore, the combination of VPA and RESV at very low doses may minimize the dose-related side effects of the drugs. These include the known VPA adverse reactions during long-term treatment, such as tremors, gastrointestinal disturbances, liver toxicity, pancreatitis and neurological disorders [33]. Furthermore, the fact that VPA and RESV impact on multiple molecular targets, including class-I HDACs, SIRT1, AMPK, and possibly sex-related pathways could reduce off-target effects.

In conclusion, several factors, including the low doses of the epigenetic drugs, their multi-target activity, and the well-known pharmacology of VPA, make the combination of VPA and RESV worthy of further investigations. Future studies on different ALS models are needed to validate this promising pharmacological treatment and to clarify its mechanism of action. An unresolved challenge in the treatment of ALS and other neurodegenerative diseases is the design of efficient drug delivery systems to increase a compound’s bioavailability, overcome biological barriers, and enhance the interaction with target sites [68,69]. The development of innovative carriers will improve the delivery of these combined compounds. Finally, a particular effort is requested to elucidate the effect of these epigenetic drugs on sex-related molecular targets in ALS. Only a better knowledge of these aspects will allow the design of personalized preventive strategies and therapies for the treatment of ALS.

## 4. Materials and Methods

### 4.1. Animals

The experiments were conducted using 44 transgenic mice (n = 22 males and n = 22 females) overexpressing human Cu/Zn superoxide dismutase 1 carrying a Gly93—Ala mutation (SOD1(G93A)) (strain designation: B6SJL-Tg(SOD1*G93A)1Gur/J 1Gur, stock number 002726), which were originally obtained from Jackson Laboratories (Ben Harbor, ME, USA). Transgenic SOD1(G93A) mice and wild-type (WT, n = 12, 6 males and 6 females) non-transgenic littermates were maintained in a B6SJL genetic background and housed under controlled environmental conditions with 12 h of light and dark cycle and received food and water ad libitum. The research complies with the commonly accepted “3Rs”. The study was conducted according to the guidelines of the Declaration of Helsinki and approved by the University of Verona committee on animal research (Centro Interdipartimentale di Servizi per la Ricerca che utilizza Animali da Laboratorio—C.I.R.S.A.L.) and by the Italian Ministry of Health (ministerial authorization number 56DC9.72, obtained on 13 August 2021). The transgenic mice were identified by polymerase chain reaction specific to human Sod1 with G93A mutation, as outlined by the Jackson Laboratories [70].

### 4.2. Disease Progression Assessment

The efficacy of drug treatment on SOD1(G93A) mice were evaluated every week, starting from postnatal day 50 to end stage of the disease. To carry out this evaluation, the animals were subjected to the Neurological score test, Paw Grip Endurance (PaGE) and rotarod test, followed by body weight evaluation every week. Each individual animal was assessed using a neurological scoring system as follows: 4 indicated that there is no sign of motor dysfunction; 3 indicated tremors in the hind limbs when mice were suspended from the tail; 2 when gait anomalies were present; 1 when the animal dragged at least one hind limb; 0 when the animal was unable to right itself within 30 s when it was placed in a supine position [31]. This method provided a rapid, non-invasive measure of disease onset and progression in SOD1(G93A) mice, in a combination of motor tests. The endstage of the disease was determined when the motor score was equal to 0 (humane endpoint).

### 4.3. Paw Grip Endurance (PaGE)

The PaGE test was used to evaluate the grip strength of the animals by positioning them in the centre of a metal grid and quickly rotated. The animals attempted this test twice for a period of 120 s each, which was set as the cut-off time with an interval of 15 min between each trial. The latency time was noted and estimated as the period spent up until the mice detached the hind limbs from the grid. The animals failed the PaGE test when they were unable to reach the cut-off time, and the onset of the disease was established from the first day the test was failed.

### 4.4. Rotarod Test

The rotarod test was used to analyze the motor coordination of the animals. The mice were placed in a rotor tube (Acceler Rota-Rod 7650, UGO BASILE, Varese, Italy) at a constant speed of 16 rpm. The temporal cut-off was set at 180 s and in case of failure, three attempts were allowed with a 5 min resting phase between each attempt. The longest latency time was recorded.

### 4.5. Drugs Treatment

Resveratrol (RESV) (554325; Merck, Milan, Italy) was dissolved in dimethyl sulfoxide (DMSO), while Sodium Valproate (VPA) (P4543; Sigma-Aldrich, Milan, Italy) was diluted in saline solution (0.9% NaCl in water). The final DMSO concentration used was approximately 0.1%. The purpose of the use of DMSO was to increase the solubility of resveratrol. Concerning RESV, the concentration of the stock solution was 15 mg/mL in DMSO (Merck, Milan, Italy) and to obtain the final concentration we performed a first dilution in saline of 1:50 from stock solution(Solution A), and then a further dilution 1:5 in saline solution from Solution A (the final solution used for the injection: 0.06 µg/µL in 50 µL, corresponding to a dose of 136 µg/kg (22 g body weight)). The concentration of a stock solution of VPA was 1 mg/mL in Ultra-Pure water (GIBCO Life Technologies, Milan, Italy) and to obtain the final concentration we performed a first dilution of 1:10 in saline from stock solution (Solution A), and a second dilution 1:5 of Solution A in saline (final solution used for the injection: 0.02 µg/µL in 50 µL, corresponding to a dose of 40 µg/kg (22 g body weight)). The drugs were prepared and combined daily (50 µL RESV + 50 µL VPA) from frozen aliquots of stock solutions and injected intraperitoneally into SOD1(G93A) mice. Groups were balanced to gender: the Vehicle (VEH) group (n = 20, 10 males and 10 females) received saline + 0.1% DMSO, at the same concentration used in the TREATED group, while the TREATED group (VPA and RESV) (n = 24, 12 males and 12 females) received 136 μg/Kg/day of RESV and 40 μg/Kg/day of VPA in combination. Treatment started on postnatal day 50 and continued daily until the animals reached the end stage of the disease. In line with the results we previously obtained treating SOD1(G93A) mice with the combination MS-275 and RESV [31], RESV was used at 136 μg/Kg/day. Keeping this RESV concentration, a VPA dose of 40 μg/Kg/day was selected on the basis of the ratio VPA:RESV we found effective in an animal model of brain ischemia [43].

### 4.6. Tissue Preparation, Staining Procedures and Stereological Count of Lumbar Spinal Cord

The end stage SOD1(G93A) mice of both the VEH (n = 10) and TREATED group (n = 12) were anesthetized with Ketamina + Xilazina + Altadol^®^ (130 + 12 + 7 mg/kg) and transcardially perfused using 0.1 M PBS followed by 4% paraformaldehyde (PFA). The spinal cord was dissected out and was further post-fixed in 4% PFA and stored overnight at 4 °C. The lumbar tract was soaked in a solution containing 30% sucrose and included in the OCT. Using a cryostat, the section was cut into 15 μm transverse slices, collected and mounted on Surgipath^®^ Apex™ upper adhesive slides (3800080E, Leica Biosystems Italia, Milan, Italy)).

For the stereological count of MN, the Nissl staining method was used. The slides were air-dried and hydrated with water for 30 s. Staining was performed by incubating the slides in a 37 °C oven for 15 min in a solution containing 0.1% cresyl violet, 92% glacial acetic acid and 8% sodium acetate. The sections were then gradually placed in increasing concentrations of ethanol (70%, 95% and 100%), cleaned with xylene, mounted with Entelan (Merck, Darmstadt, Germany) and covered with a coverslip. The MN of the lumbar tract (L1-L5) were counted using a computer-assisted microscope (Olympus BX6 with Retiga 2000R camera) with the Stereo investigator software (MicroBrightField, Williston, VT, USA) at 40× magnification. Cells with nucleoli on the plane of focus, size and shape typical of MN were counted. The values from the sections were computed for the summation, and the mean number was then computed from the average number derived from each animal.

To investigate gliosis activation to detect microglia cells in the lumbar tract of the spinal cord, immunohistochemistry for light microscopy was performed. The sections were incubated for 15 min in 1% H_2_O_2_ to quench endogenous peroxidase and preincubated for 1 h in 5% of Normal Goat Serum (NGS) and 0.3% Triton X-100 in PBS. The slides were then incubated overnight in mouse anti-mouse Iba1 antibody (1:500, 019–19741, Fujifilm WAKO, Osaka, Japan) in 1% NGS and 0.3% Triton X-100 in PBS. The sections were rinsed in water 3 times for 5 min each and incubated for 1h in biotinylated goat anti-mouse IgG (1:200, BA-9500, Vector Laboratories, Burlingame, CA, United States) in 1% NGS and 0.3% Triton X-100 in PBS. The reaction was developed with the avidin-biotin-peroxidase kit (ABC kit; Vector) using 3–3′-diaminobenzidine as chromogen. The sections were dehydrated through increasing grades of ethanol (70%, 95% and 100%), cleared in xylene, and cover-slipped with Entellan. The microglial cells of the lumbar tract (L1-L5) were analyzed using a computer-assisted microscope (Olympus BX6 with Retiga 2000R camera) with the Stereo investigator software (MicroBrightField, Williston, VT, USA).

### 4.7. Immunofluorescence Procedures

The immunofluorescence procedure was performed on lumbar spinal cord MN of end stage SOD1(G93A) of both VEH (n = 11) and TREATED (n = 10) groups, and age and sex-matched WT (120–140 days, n = 4) to investigate the acetylation state of the Lys9 of H3 and SMI-32. The animals were deeply anesthetized and transcardially perfused, as described above. First, the slides were incubated for 1 h in 2.5% NGS and 0.3% Triton X-100 diluted in PBS. Then the slides were incubated overnight in H3AcK9 (1:250, GeneTex, GTX88007) and SMI-32 (1:1000, Biolegend, #SMI-32P) antibodies in 1.25% NGS and 0.3% Triton X-100 diluted PBS. The slides were rinsed and the sections were incubated for 1 h in goat anti-rabbit 594 IgG (1:1000, A11012, ThermoFisher Scientific, Milan, Italy) and goat anti-mouse IgG 488 secondary (1:1000, A32723, ThermoFisher Scientific, Milan, Italy) antibodies in 1% GS and 0.3% Triton X-100 in PBS. The nuclei were counterstained with DAPI (4′,6-Diamidino-2-Phenylindole, Dihydrochloride) (1:2000, D1306 ThermoFisher Scientific, Milan, Italy) for 5 min and washed in PBS. The sections were then coverslipped with Fluorescent Mounting Medium (S3025, Dako-Agilent, Santa Clara, CA, USA), and the immunofluorescence was analyzed with a TCS-SP5 confocal microscope (Leica-Microsystems, Wetzlar, Germany), in a dual-channel acquisition setup, using UV, 488 nm and 543 nm excitation beams.

### 4.8. Immunoprecipitation and Western Blot

Fresh lumbar spinal cords were collected from SOD1(G93A) (n = 21) and WT (age-matched, n = 6) mice after cervical dislocation. To investigate the RelA acetylation state, we performed an immunoprecipitation assay, while the expression of an enzyme involved in RelA acetylation (AMPK), anti-apoptotic (Bcl-xL) and neurotrophic factor (BDNF) was achieved by Western blot assays. The proteins of the nuclear and cytoplasmic fraction were obtained using NE-PER™ Nuclear and Cytoplasmic Extraction Reagents (ThermoFisher Scientific, Milan, Italy) according to the manufacturer’s instructions. Protein concentration was determined using the Pierce™ Detergent Compatible Bradford Assay (23236, Thermo Fisher Scientific, Milan, Italy). Fifty micrograms of nuclear protein fraction were incubated with goat anti-RelA (10 µg/mg lysate, LS-C290611-100) overnight at 4 °C on a rotor plate. The RelA protein associated with the antibodies were precipitated with Protein A-Sepharose (CL-4B, 17-0780-01, GE Healthcare Bio-Sciences, Uppeala, Sweden) according to the user’s manual. After washing, the immunoprecipitated proteins were resolved by 4–20% SDS-polyacrylamide gel (Biorad, Segrate, Milan, Italy) and transferred to polyvinylidene difluoride (PVDF) membrane. The following antibodies were used to detect the immunoprecipitated proteins: rabbit anti-NF-kB p65 (1:1000, GTX107678, GeneTex San Antonio, TX, USA), rabbit anti-Acetyl Lysine (1:500, AB3879, Merck Millipore, Milan, Italy) and rabbit anti-NF-kB p65 (acetyl lys 310) (2.5 µg/mL, ab19870, Abcam, Cambridge, United Kingdom). Horseradish peroxidase (HRP) polyclonal goat anti-rabbit immunoglobulins (1:2000, #P0448, Dako, Santa Clara, CA, USA) were used to detect primary antibodies.

Thirty micrograms of cytoplasmic protein fraction were separated on 4–20% SDS-polyacrylamide gel and transferred onto the PVDF membrane. Primary antibodies were used at 1:1000 dilution and appropriate HRP-conjugated secondary antibodies were used at 1:2000 dilution in 5% BSA and PBS-Tween 0.1% blocking solution. For the primary antibodies against murine AMPKα rabbit mAb (#5831, Cell Signaling) and p-AMPKα (Tr172) rabbit mAb (#2535, Cell Signaling), rabbit anti- Bcl-xL (#2762, Cell Signaling), mouse anti-BDNF (ab203573, Abcam) and a mouse monoclonal anti-GAPDH (AM4300, ThermoFisher Scientific, Milan, Italy) were used. All membranes were then incubated with a chemiluminescent HRP substrate (WBKLS0500, Merck Millipore, Milan, Italy) and detected with G:BOX F3 GeneSys (Syngene, Cambridge, United Kingdom).

### 4.9. Data Analysis and Statistics

The data obtained from the motor tests were analyzed using the two-way ANOVA followed by Bonferroni multiple comparison tests. Data are expressed as mean ± standard error of the mean (SEM). Regarding data of the disease onset and the survival, the Log-rank test (Mantel–Cox) was used. Data are expressed as a mean ± SEM. Concerning the stereological count of MN, a two-tailed Student’s T was used. Data are reported as mean ± SEM. GraphPad Prism 8 software was used for statistical analysis, and graphs and significance were accepted at *p* < 0.05. Densitometric analysis of Western blot was performed with Fiji (ImageJ) software open source [70] and the stereological MN count data were analyzed using the one-way ANOVA, followed by Tukey’s multiple for comparisons tests. Data are reported as mean ± SEM. For all statistical analysis and graphs, GraphPad Prism 8 Software was used, and the significance was accepted at *p* < 0.05.

## Data Availability

Not applicable.

## Supplementary Materials

The following supporting information can be downloaded at: https://www.mdpi.com/article/10.3390/ijms23031047/s1.
