# Supplementary material for: Beneficial and Sexually Dimorphic Response to Combined HDAC Inhibitor Valproate and AMPK/SIRT1 Pathway Activator Resveratrol in the Treatment of ALS Mice
Source: Int J Mol Sci. 2022 Jan 19;23(3):1047. doi: 10.3390/ijms23031047 (PMC8835218; doi:10.3390/ijms23031047)
Supplement: Supplementary file 1 [file ijms-23-01047-s001.zip › supplementary data 1.pdf]

Beneficial and Dimorphic Response to Combined HDAC Inhibitor Valproate and AMPK/SIRT1 Pathway Activator Resveratrol in the Treatment of ALS Mice

Oluwamolakun Bankole [1,†], Ilaria Scambi [1,†], Edoardo Parrella [2], Matilde Muccilli [1], Roberta Bonafede [1], Ermanna Turano [1], Marina Pizzi [2,*] and Raffaella Mariotti [1,*]

[1] Department of Neurosciences, Biomedicine and Movement Sciences, University of Verona, 37134 Verona, Italy; oluwamolakunoluwatobi.bankole@univr.it (O.B.); ilaria.scambi@univr.it (I.S.); matilde.muccilli@univr.it (M.M.); roberta.bonafede@yahoo.com (R.B.); ermanna.turano@univr.it (E.T.)
[2] Department of Molecular and Translational Medicine, Division of Pharmacology, University of Brescia, 25123 Brescia, Italy; e.parrella@unibs.it
* Correspondence: marina.pizzi@unibs.it (M.P.); raffaella.mariotti@univr.it (R.M.); Tel.: +39-030-371-7501 (M.P.); +39-045-802-7164 (R.M.)
† These authors contributed equally to this work.

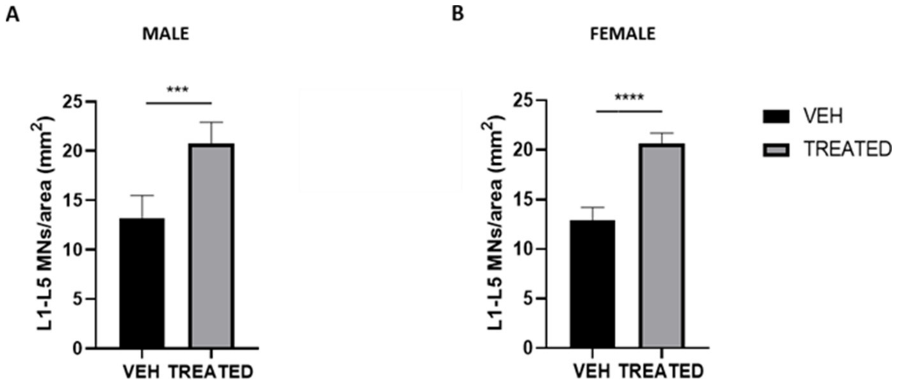

Figure S1. Motor neuron (MN) count in the lumbar tract of the spinal cord (L1–L5) of male and female SOD1(G93A) mice: (**A**) In the male mice, a significant increase in MN count was observed in the TREATED group with respect to the VEH (p = 0.0004; n = 5 VEH, 6 TREATED); (**B**) Similar to the male SOD1(G93A) mice, the female mice showed a significant increase in MN count in the L1-L5 segment of the TREATED group with respect to the VEH ($p < 0.0001$; n = 5 VEH, 6 TREATED). Data were analyzed by Student *t*-test, expressed as mean ± SEM.*** $p < 0.0005$; and **** $p < 0.0001$.

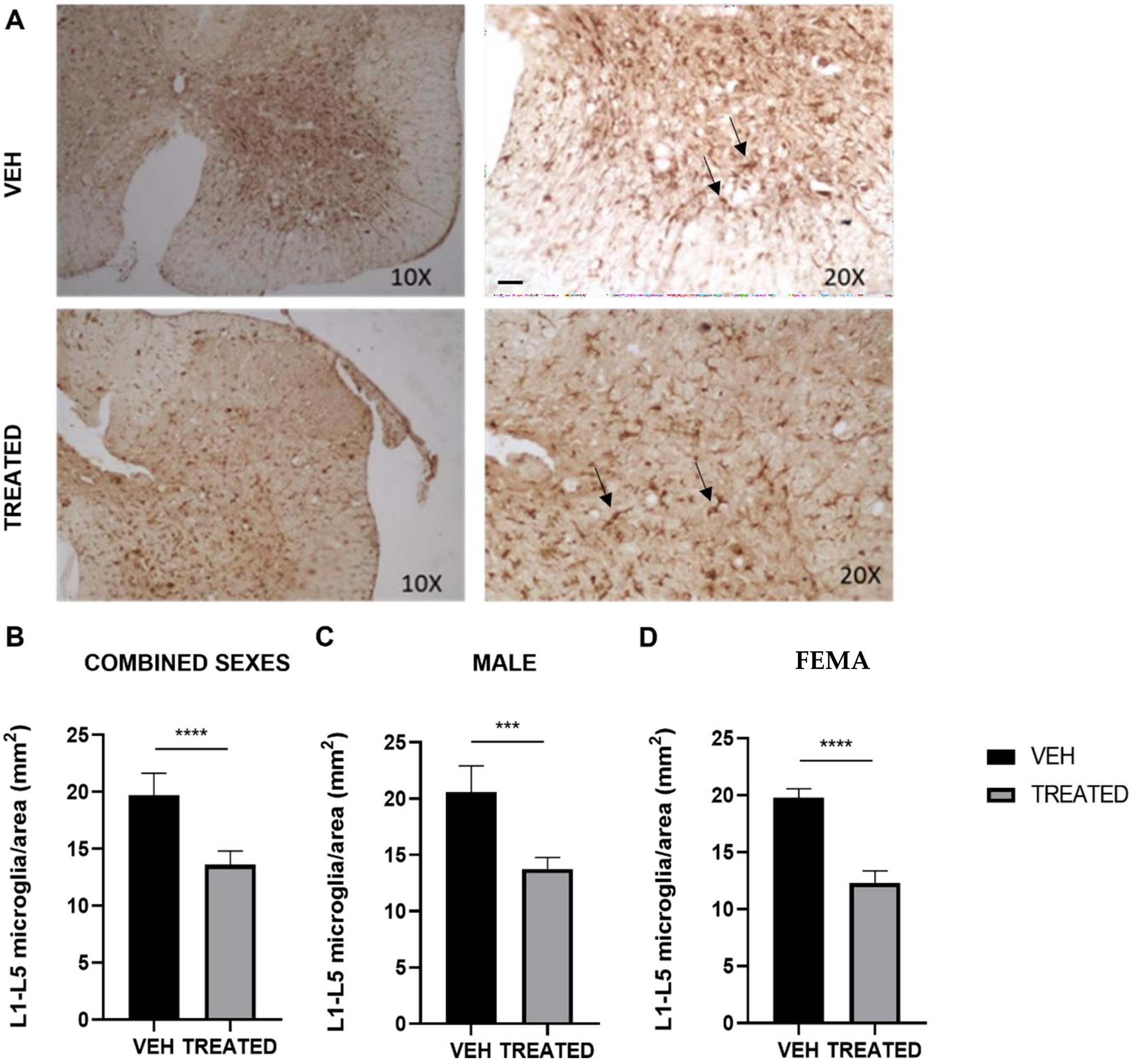

Figure S2. Microglia count in the lumbar tract of the spinal cord (L1–L5) of combined sex, male and female SOD1(G93A) mice: (**A**) Image showing Iba1 positive cells in the L5 segment of VEH and TREATED SOD1(G93A) mice. The arrows indicate visible microglia; (**B**) The graph shows the number of Iba1 positive cells in the lumbar tract of combined sex. A significant decrease (31%) in microglia count was observed in the TREATED group compared to the VEH ($p < 0.0001$; n = 10 VEH, 12 TREATED); (**C**) In the male mice, there was a significant decrease in microglia count (33%) in TREATED compared to the VEH ($p = 0.0001$; n = 5 VEH, 6 TREATED); (**D**) Female mice also showed a significant decrease (38%) in the number of Iba1 positive cells in the L1-L5 tract in the TREATED with respect to the VEH, ($p < 0.0001$; n = 5 VEH, 6 TREATED). Images on the left panel have magnification 10×, while images on the right panel have magnification 20×, scale bar 100 µm. Data were analyzed by Student *t*-test, expressed as mean ± SEM. *** $p < 0.0005$ and **** $p < 0.0001$.

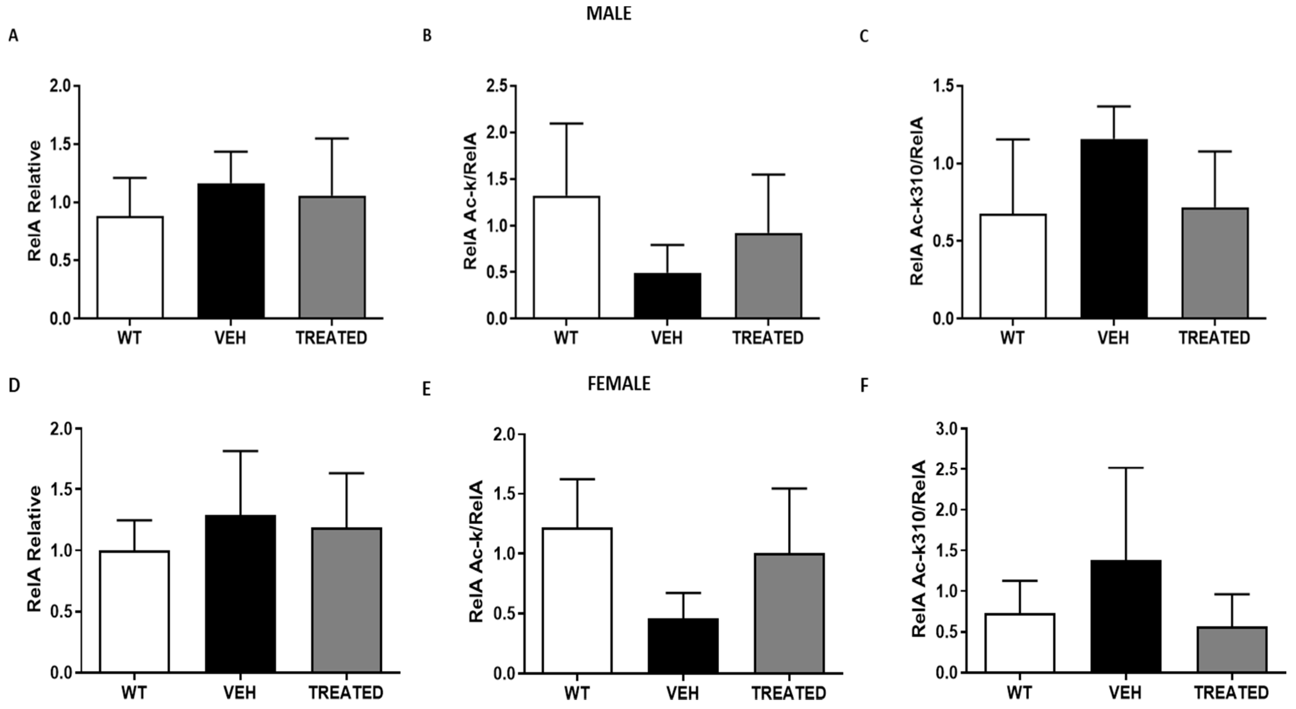

Figure S3. Immunoprecipitation of RelA subunits in male and female SOD1(G93A) mice: (**A**) An increase in RelA expression in the VEH group of male mice was observed compared to the WT and TREATED animals without statistically significance; (**B**) A reduction was observed in total lysine (RelA Ac-k) acetylation in the VEH group compared to the WT. This was accompanied by an increase in RelA Ac-k acetylation of male animals in the TREATED group without statistical significance; (**C**) Furthermore, acetylation of the specific RelA Ac-k310 was increased in the VEH group compared to the WT, while the treatment led to a decrease in acetylation with respect to the VEH without significance. (n = 3 WT, 5 VEH, 6 TREATED); (**D**) In female mice, densitometric analysis shows an increase in RelA expression in the VEH group compared to the WT and TREATED group without statistical significance; (**E**) RelA Ac-k acetylation was reduced in the VEH group of compared to the WT while the acetylation was increased in the TREATED group but without any significance; (**F**) An increase was observed in the acetylation at K310 in the VEH group of femle mice compared to the WT, while the treatment decreased the RelA K310 acetylation compared to the VEH. This was however, not statistically significant (n = 3 WT, 5 VEH, 6 TREATED). Results were analyzed by one-way ANOVA followed by Tukey's multiple comparisons test. Graphs are shown as mean ± SEM.

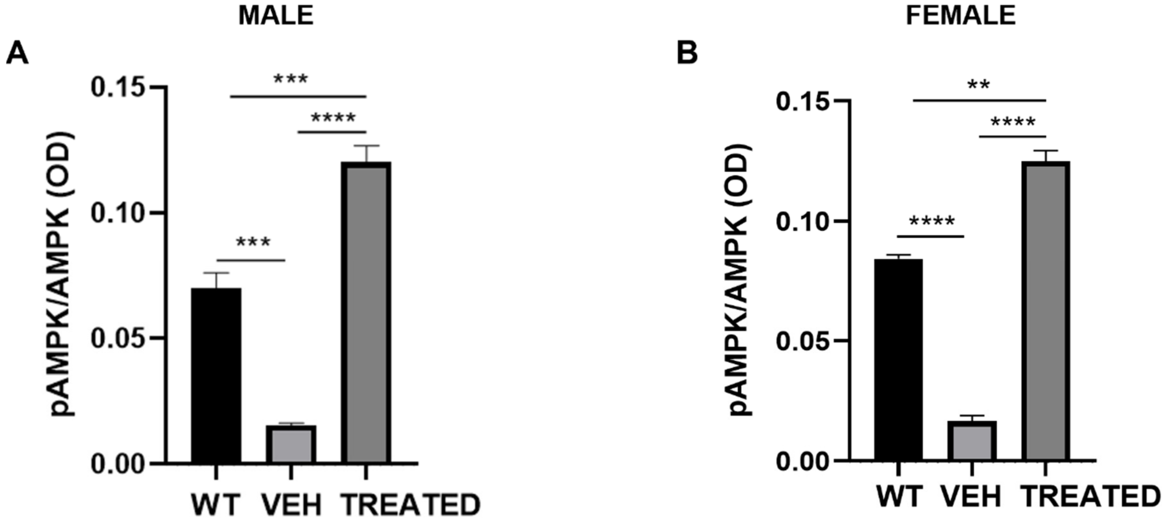

Figure S4. Analysis of phosphorylation state of APMK in male and female SOD1(G93A) mice: (**A**) Phosphorylation state of AMPK male mice was reduced in the VEH compared to the WT with a significance of ($p = 0.0002$). Animals in the TREATED group showed an increased phosphorylation state with a significance of ($p < 0.0001$) with respect to the VEH. A significant increase was also observed in the TREATED mice compared to the WT ($p = 0.0003$; n = 3 WT, 5 VEH, 6 TREATED); (**B**) In female mice, a significant reduction ($p < 0.0001$)

was observed in the phosphorylation state of AMPK in the VEH group compared to the WT. TREATED mice also displayed increased phosphorylation compared to the VEH with a significance of (p<0.0001). Furthermore, there was a significant increase in the phosphorylation state of TREATED animals compared to the WT (p = 0.0010; n = 3 WT, 5 VEH, 6 TREATED). Results were analyzed by one-way ANOVA followed by Tukey's multiple comparisons test. Graphs are shown as mean ± SEM. ** $p < 0.005$; *** $p < 0.0005$; and **** $p < 0.0001$.

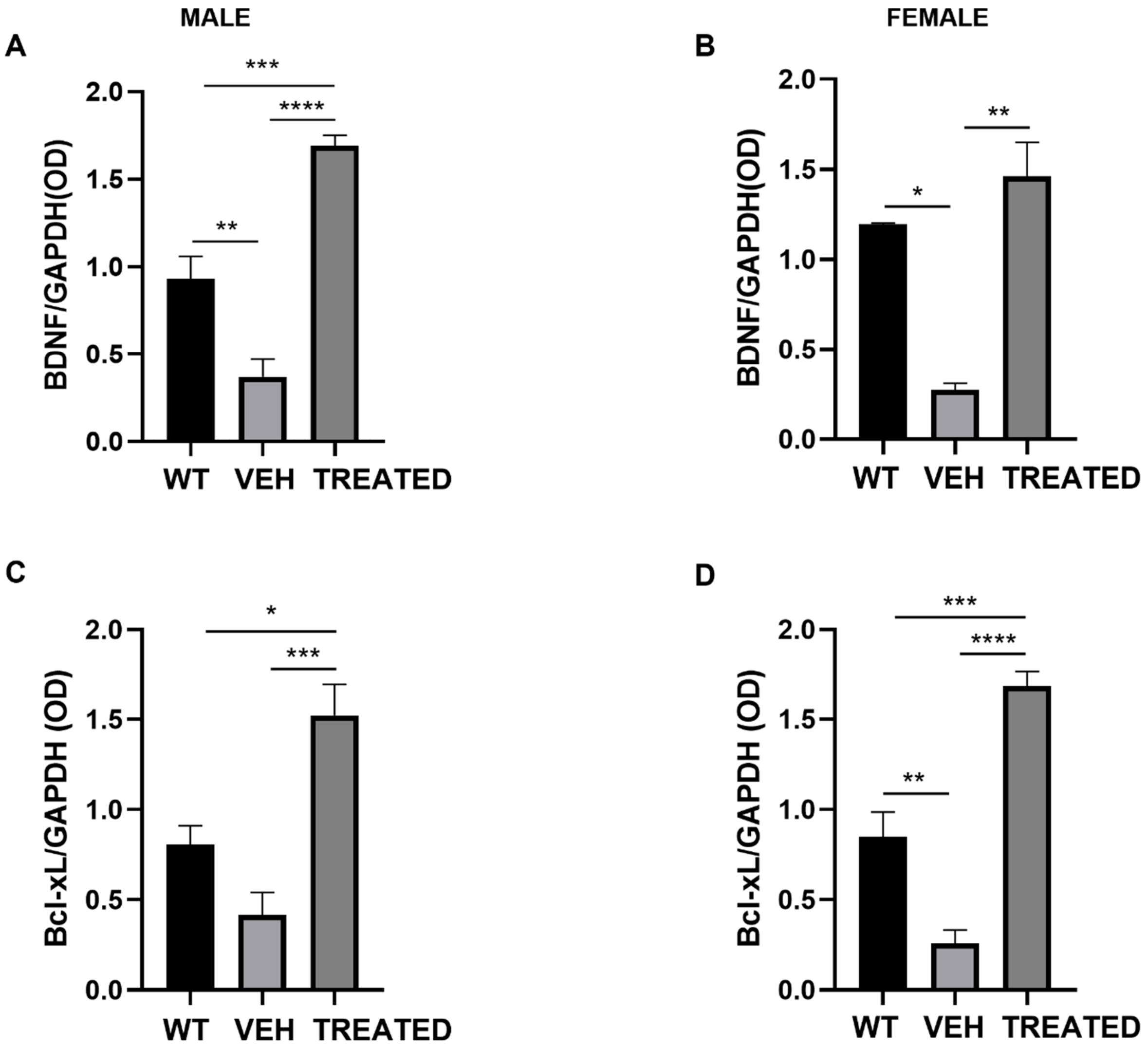

Figure S5. Analysis of neurotropic factor BDNF and anti-apoptotic factor Bcl-xL male and female SOD1(G93A) mice: (**A**) Expression level of BDNF in male mice was significantly reduced (p = 0.0049) in the VEH compared to the WT. TREATED mice displayed an increase in BDNF with respect VEH with a significance of ($p < 0.0001$). Similarly, a significant increase was observed in the VEH group compared to the WT, (p = 0.0003; n = 3 WT, 5 VEH, 6 TREATED); (**B**) The neurotropic factor BDNF in female animals was reduced in VEH mice compared to the WT with significance of (p = 0.0365). The treatment led to an increased expression of BDNF with statistical significance of (p = 0.0055). No significance was observed between WT and TREATED group (p = 0.6878; n = 3 WT, 5 VEH, 6 TREATED); (**C**) In male animals, the anti-apoptotic protein was reduced in VEH animals compared to the WT without any significance (p = 0.3497).Treatment with the epigenetic drugs resulted in an increased expression of Bcl- xL with a significance of (p = 0.0007).Likewise, a significant increase was observed in the Bcl-xL expression of the treated animals compared to the WT(p = 0.0400); (**D**) The anti-apoptotic factor Bcl-xL was significantly reduced in the VEH female animals compared to the WT (p = 0.0097). In the TREATED mice, a drastic increase ($p < 0.0001$) in the expression of the protein was observed with respect to the VEH Lastly, the treated animals also displayed a.significant increase compared the WT (p = 0.0009). Results were analyzed by one-way ANOVA followed by Tukey's multiple comparisons test. Graphs are shown as mean ± SEM. * $p < 0.05$; ** $p < 0.005$; *** $p < 0.0005$ and **** $p < 0.0001$.

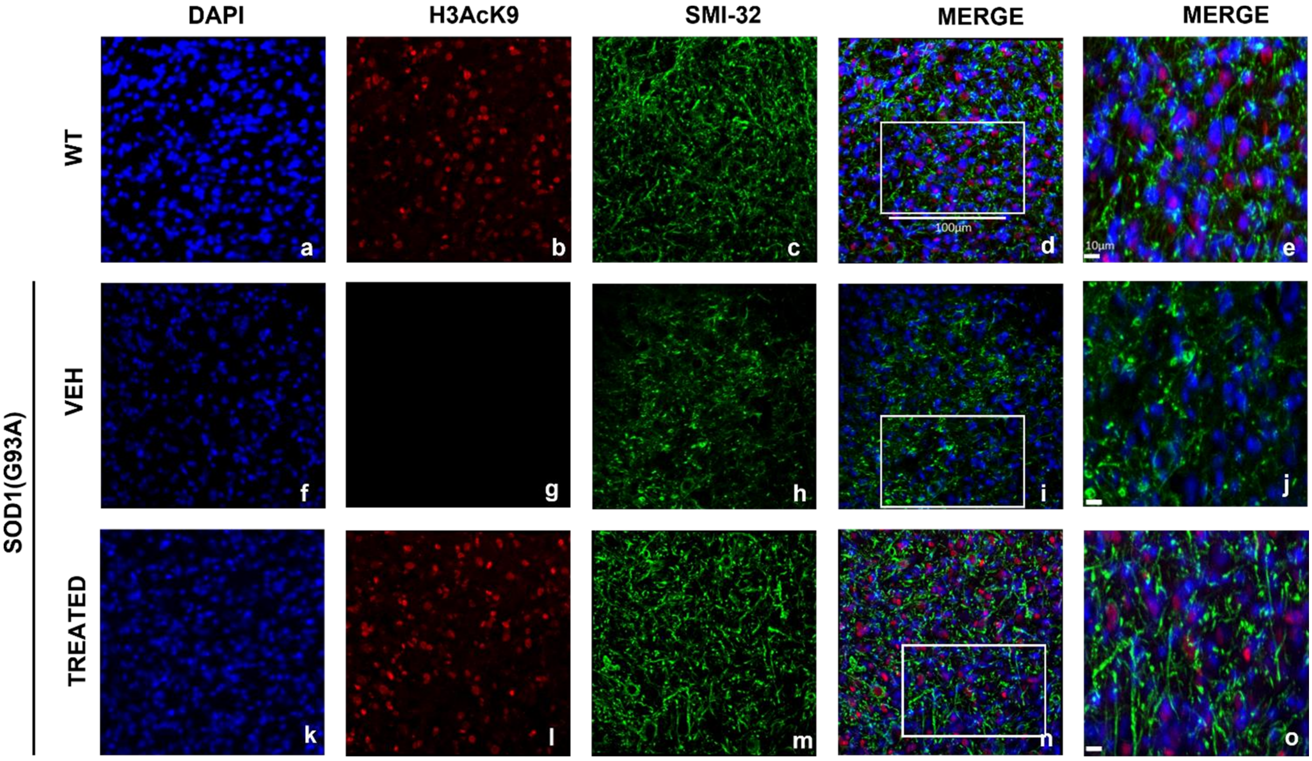

Figure S6. Histone 3 acetylation in the lumbar spinal cord of female WT and SOD1(G93A) mice: The figure panel shows the different acetylation state of lysine 9 of histone 3 in the lumbar spinal cord of wild type (WT) mice, untreated (VEH) and treated SOD1(G93A) groups. The nuclei were stained in blue with DAPI (**a**,**f**,**k**).The acetylation state of histone 3 was identified by H3AcK9 antibody in red (**b**,**g**,**l**), while the motor neurons (MN) was detected by identifying the antibody neurofilament H with the SMI-32 antibody in green (**c**,**h**,**m**). The acetylation state of histone 3 was drastically reduced in the VEH group (**g**) compared to WT animals (**b**). The treatment with the epigenetic drugs led to a restoration of the acetylation of histone 3 in the treated group (**l**), (n = 3 WT 5 VEH, 6 TREATED). Figure **d**, **i**, **n** shows the superimposed images of DAPI, SMI-32 and H3AcK9 in the WT, VEH, and treated groups respectively. Magnification 20×, scale bar 100 μm (**a**–**d**, **f**–**i** and **k**–**n**). The figure panel **e**, **j**, **o** shows the highlighted area in **d**, **i**, **n** at higher magnification 40×, scale bar 10 μm.
