# Supplementary material for: Beneficial and Sexually Dimorphic Response to Combined HDAC Inhibitor Valproate and AMPK/SIRT1 Pathway Activator Resveratrol in the Treatment of ALS Mice
Source: Int J Mol Sci. 2022 Jan 19;23(3):1047. doi: 10.3390/ijms23031047 (PMC8835218; doi:10.3390/ijms23031047)
Supplement: Supplementary file 1 [file ijms-23-01047-s001.zip › supplementary data 2.pdf]

Beneficial and dimorphic response to Combined HDAC Inhibitor Valproate and AMPK/SIRT1 Pathway Activator Resveratrol in the Treatment of ALS Mice

Oluwamolakun Bankole [1,+], Ilaria Scambi [1,+], Edoardo Parrella [2], Matilde Muccilli [1], Roberta Bonafede [1], Ermanna Turano [1], Marina Pizzi [2,*] and Raffaella Mariotti [1,*]

[1] Department of Neurosciences, Biomedicine and Movement Sciences, University of Verona, 37134 Verona, Italy; oluwamolakunoluwatobi.bankole@univr.it (O.B.); ilaria.scambi@univr.it (I.S.); matilde.muccilli@univr.it (M.M.); roberta.bonafede@yahoo.com (R.B.); ermanna.turano@univr.it (E.T.)

[2] Division of Pharmacology, Department of Molecular and Translational Medicine, University of Brescia, 25123 Brescia, Italy; e.parrella@unibs.it (E.P.)

* Correspondence: raffaella.mariotti@univr.it (R.M.) Tel.: +39-045-802-7164 and marina.pizzi@unibs.it (M.P.) Tel.: +39-030-371-7501

*Authors to whom correspondence should be addressed.

+ These authors contributed equally to this work.

Supplementary figure 7

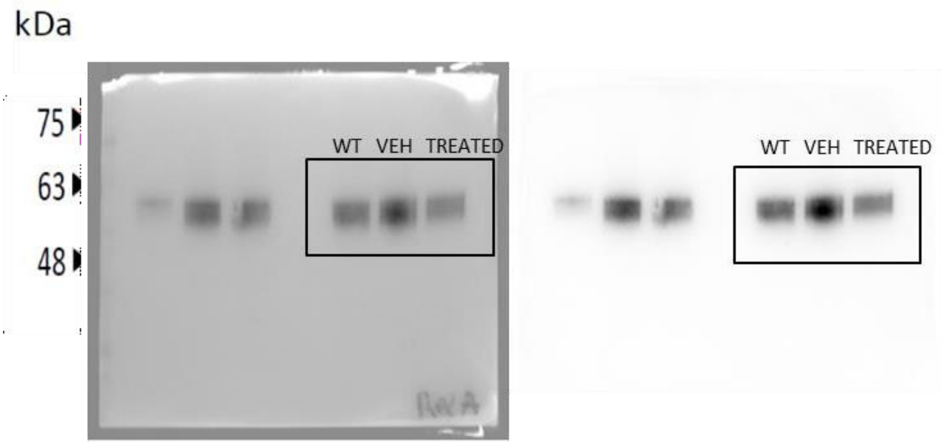

Blot 1: RelA

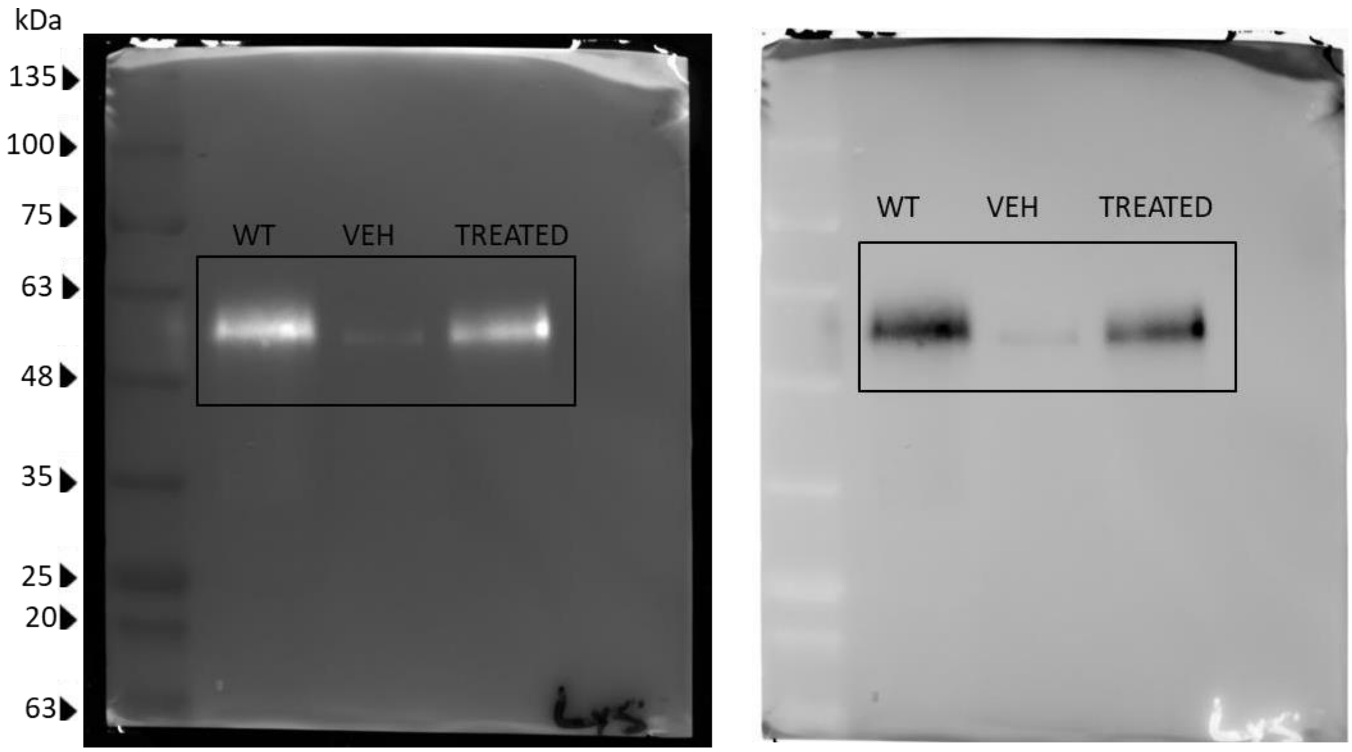

Blot 2: Ac-RelA (tot Lys)

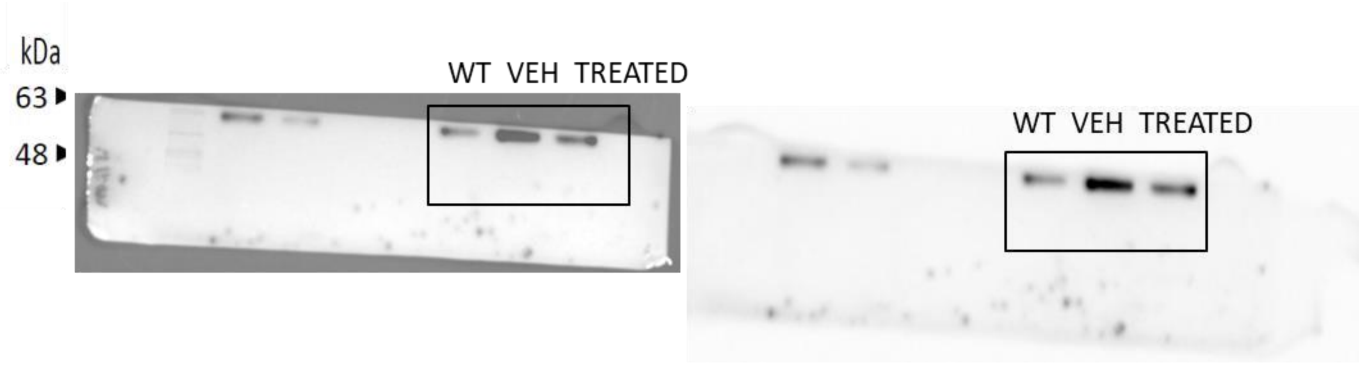

Blot 3. Ac-RelA (Lys 310)

Figure S7: Western blot of RelA subunit of the nuclear fraction isolated from lumbar spinal cord of WT, VEH and TREATED groups. Blot 1 – RelA, blot 2 – Ac-RelA (tot lys) and Blot 3 – Ac-RelA (Lys 310) membrane are shown merged with the protein molecular weight marker (kDa) on the left, on the right are shown the full length western blots of gels presented in Figure 5a of the main article. The black line boxes referred to the cropped parts that are showed in the main article.

Supplementary figure 8

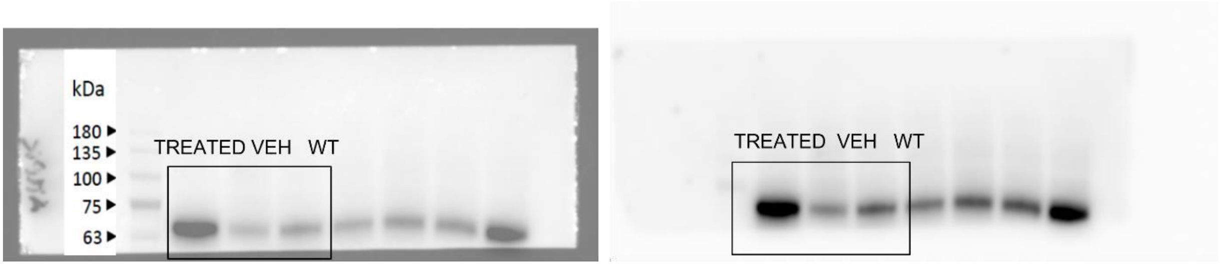

Blot 1: pAMPK

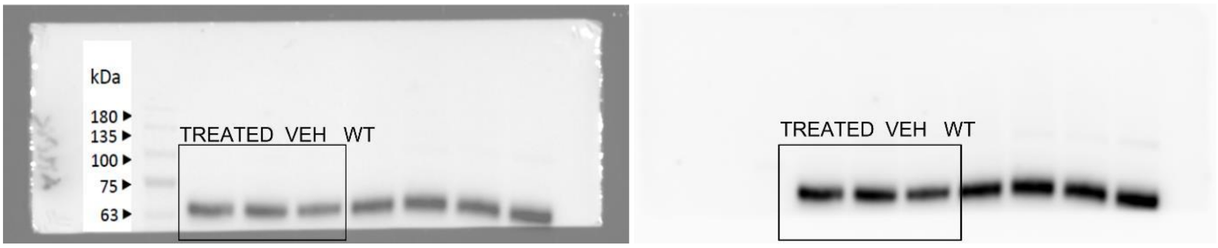

Blot 2: AMPK

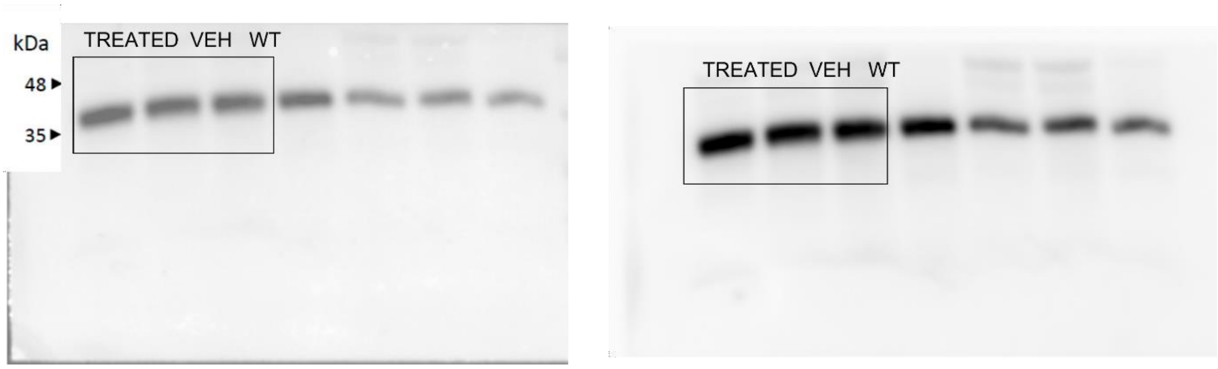

Blot 3: GAPDH

Figure S8. Western blot of cytoplasmic fraction isolated from lumbar spinal cord of WT, VEH, TREATED groups probed with pAMPK (62kDa), AMPK (62kDa) and GAPDH (37kDa) antibodies: The figure shows western blots of gels shown in Figure 6a of the main article. The black-lines boxes on the images refer to the cropped part presented in the main article and the molecular weight marker of each protein is indicated on the left (kDa).

Supplementary figure 9.

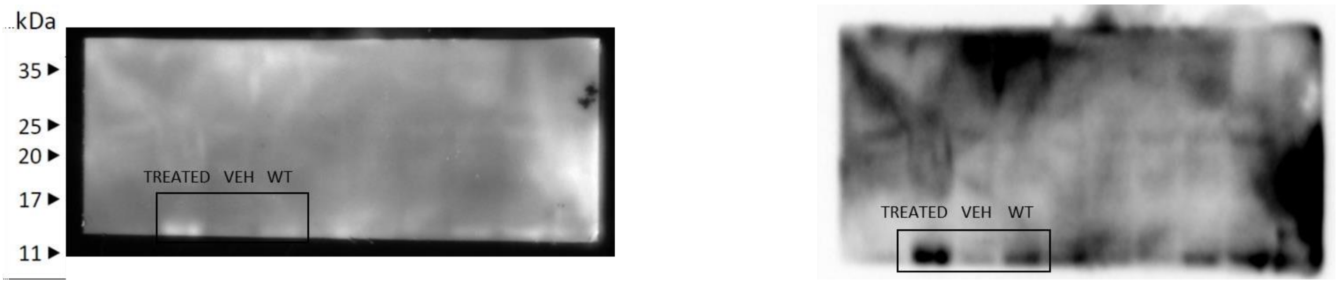

Blot 1. BDNF

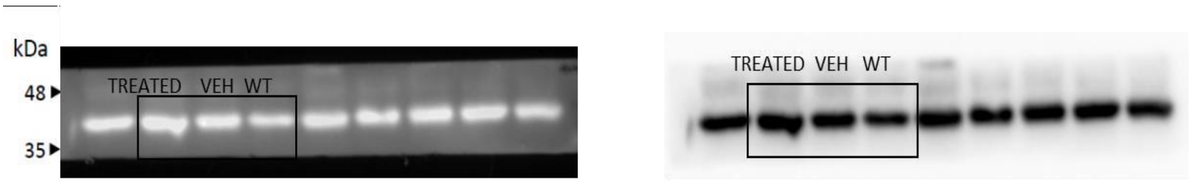

Blot 2. GAPDH for BDNF

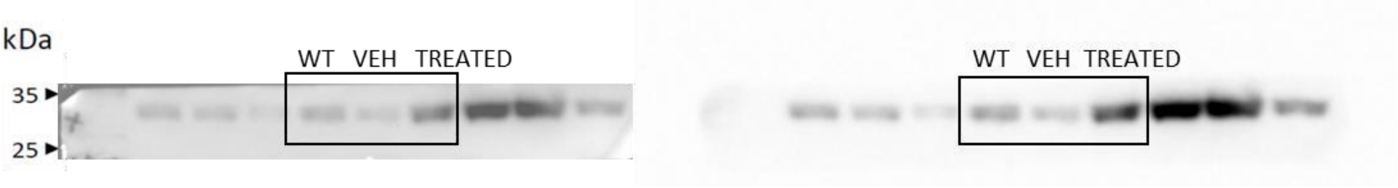

Blot 3. Bcl-xL

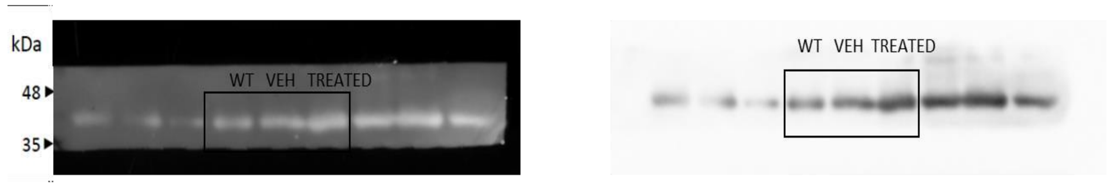

Blot 4. GAPDH for Bcl-xL

Figure S9. Western blot of cytoplasmic fraction isolated from lumbar spinal cord of WT, VEHand TREATED mice probed with Bcl-xL (30 kDa), BDNF (14 kDa) and GAPDH (37 kDa) antibodies. The figure shows full length western blots of gels presented in Figure 7A (BDNF) and Figure 7C (Bcl-xL) of the main article. The boxes on the images highlight the cropped parts that are showed in the main article. The protein molecular weight marker (kDa) is indicated on the left.
